# Crystallization Behavior and Morphology of Biodegradable Poly(ε-caprolactone)/Reduced Graphene Oxide Scaffolds

**DOI:** 10.3390/biomimetics7030116

**Published:** 2022-08-24

**Authors:** Esperanza Díaz, Ainhoa Mendivil, Joseba León

**Affiliations:** 1 Escuela de Ingeniería de Bilbao, Departamento de Ingeniería Minera, Metalúrgica y Ciencia de Materiales, Universidad del País Vasco (UPV/EHU), 48920 Portugalete, Spain; 2 BCMaterials—Basque Centre for Materials, Applications and Nanostructures, UPV/EHU Science Park, 48940 Leioa, Spain

**Keywords:** PCL, rGO, scaffolds, morphology, non-isothermal crystallization

## Abstract

Morphology, thermal properties and the non-isothermal melt crystallization kinetics of biodegradable poly(ε-caprolactone) (PCL)/reduced graphene oxide (rGO) scaffolds are studied with differential scanning calorimetry (DSC) at various cooling rates (5, 10, 15 and 20 °C/min). Thermally induced phase separation was used to manufacture the scaffolds (TIPS). The micrographs show a more homogeneous and defined morphology with larger pores and thicker pore walls. The melting temperature (Tm), melting enthalpy (ΔHm), crystallization enthalpy (ΔHc) and degree of crystallinity (Xc) increased with the addition of rGO, suggesting larger and more perfect crystalline structures. The degree of crystallinity increased with the presence of rGO. The crystallization peak shifted to higher temperatures as the rGO concentration increased independently of the cooling rates. The peak shifted to lower temperatures as the cooling rate increased with the same rGO composition. The values of t_1/2_ (time needed to reach 50% crystallization) were lower for scaffolds with rGO. The values of the crystallization rate coefficient were higher when the porous support contained rGO, which indicates that their crystallization systems are faster. The activation energy obtained with the Kissinger method decreased with the presence of rGO. The results indicate that reduced graphene oxide acts as a nucleating agent in the non-isothermal melt crystallization process. The addition of small quantities of rGO changes their thermal properties with which they can be modified for application in the field of tissue engineering.

## 1. Introduction

Poly(ε-caprolactone) (PCL) is a biodegradable and biocompatible polyester aliphatic that is widely used in the field of tissue regeneration, due to its good physical and biological properties [1,2]. Its melting temperature of 60–64 °C is higher than that of the human body, and its Tg is −60 °C so it is in a semi-crystalline form whenever in the human body due to its regular structure [3]. The higher its molecular weight, the more difficult it is for the polymeric chains to arrange themselves, and therefore the lower its crystallinity.

Both hydrolytic mechanisms and microorganisms degrade PCL under certain physiological conditions, which is why PCL is often found in packaging materials [4]. PCL is one of the slowest aliphatic polyesters to degrade with a degradation time that is usually over one year, due to the presence of five hydrophobic groups, -CH_2_, in its repetitive units [5]. On the other hand, its mechanical properties are not sufficient for applications in bone regeneration processes, which is why it is usually combined with different reinforcements, such as nHA [6], MWCNT [7,8], GO [9] and rGO [10].

Graphene, one of the allotropic forms of carbon, was first isolated in 2004. It is a two-dimensional material, where each carbon atom forms sp^2^ bonds with three other carbon atoms, creating a flat sheet with a honeycomb-like structure that has high conductivity and high mechanical planar strength [11]. When this graphene is grouped in an indefinite number of layers, a material known as graphite is obtained, from which graphite oxide can be generated and, in turn, graphene derivatives: graphene oxide and reduced graphene oxide [11,12]. Reduced graphene oxide (rGO) is produced by removing the oxygen content of the graphene oxide structure through chemical or thermal processes [11,13]. rGo is a very hard material, an excellent conductor of electric currents and is highly malleable, a property that is very rare among hard materials that are often of low malleability and highly brittle. Its malleability opens up a whole world of applications for this material. The most outstanding applications of graphene oxide are in composite materials and organic solvents, as it easily disperses in water and contains modifiable oxygenated groups.

The thermal history of polymers influences their crystalline structures, which in turn influences their physical properties, biodegradability and morphology; hence, the importance of studying crystallization processes in the research [14]. In general, the crystallinity of aliphatic polyesters is not very significant, especially in non-isothermal conditions, such as extrusion and injection molding. This limits their development and practical application [15]. Different nucleation agents have been added to accelerate the crystallization rate of some of these semi-crystalline aliphatic polyesters, among which are hydroxyapatite [16,17], montmorillonite [18], carbon nanotubes [8,15] and GO [19].

In the present study, thermally induced phase separation (TIPS) is used to manufacture biodegradable and biocompatible PCL/rGO nanocomposite scaffolds, with different rGO contents of 0–1%. The morphology, thermal properties and non-isothermal melt crystallization are studied to evaluate the effect of rGO. We use some existing models for which the nucleation effect of rGO, the activation energy of the process and the crystallization kinetics were previously ascertained.

## 2. Experimental

### 2.1. Materials and Scaffold Preparation

**Figure 1:**
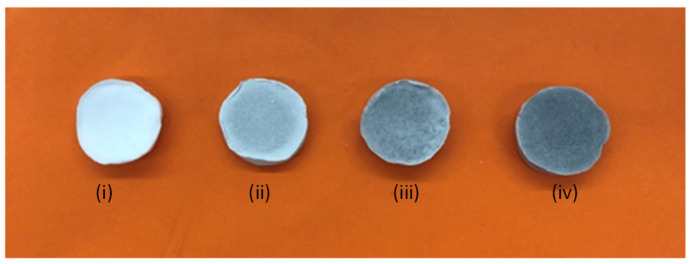
From left to right: (**i**) PCL; (**ii**) PCL/0.3% rGO; (**iii**) PCL/0.6% rGO and (**iv**) PCL/1% rGO.

Medical grade Poly(ε-caprolactone) was supplied from Aldrich (Barcelona, Spain). Gel permeation chromatography (GPC, Perkin Elmer 200, Triad Scientific, Manasquan, NJ, USA) was used to calculate the average molecular weight: Mn = 45,000. A solution of PCL in 1, 4 dioxane (2.5% *w*/*v*) was stirred at 50 °C for 2 h to obtain a homogeneous polymer solution. The mixture was mixed with reduced graphene oxide (Graphenea, San Sebastián, Spain) and dispersed by sonication. The polymer solutions were then poured into aluminum molds specially built for the manufacture of these scaffolds and chilled to −60 °C. The samples were frozen freeze-dried at −62 °C and 0.5 mmHg for 11 days to remove the solvent (lyophilization); the resulting scaffolds (Figure 1) had a porosity of over 90%. The porosity of the scaffolds was quantified by mercury pycnometry [6]. The solvent, 1,4-dioxane, (Panreac p.a. Barcelona, Spain), was distilled using conventional methods.

### 2.2. Characterizations

Scanning electron microscopy (SEM) (HITACHI S-4800, Tokyo, Japan) was used to perform PCL and PCL/rGO micrographs of the scaffolds. The scaffolds were sputtered with a gold coating using a JEL Ion Sputter JFC-1100 (Amiron Machinery, Oxnard, CA, USA) at 1200 V and 5 mA.

A Q-200 TA instruments calorimeter (Water, Raleigh, NC, USA) with universal analysis was used for obtaining differential scanning calorimetry measurements. The weights of the samples varied between 4 and 6 mg. All operations were performed in a nitrogen atmosphere. For non-isothermal crystallizations, the samples were heated from room temperature to 100 °C and then cooled to 0 °C at various cooling rates: 5, 10, 15 and 20 °C/min. The melting temperature (Tm) was determined from the endothermic melting peak position, the area under the melting peak was calculated to obtain the melting enthalpy (ΔHm), and the area under the crystallization peak was calculated to obtain the crystallization enthalpy (ΔHc).

## 3. Results and Discussion

### 3.1. Morphology

**Figure 2:**
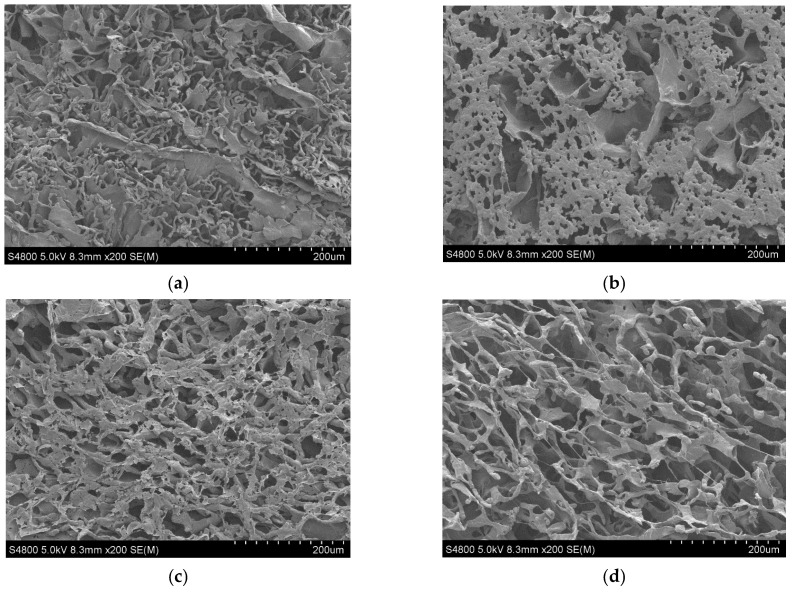
SEM micrographs × 200 of PCL nanocomposite scaffolds: (**a**) PCL; (**b**) PCL/0.3% rGO; (**c**) PCL/0.6% rGO; (**d**) PCL/1% rGO.

The micrographs of PCL and its composite scaffolds with rGO are presented in Figure 2. As we can observe, the addition of rGO produced a more homogeneous and well-defined structure with pores that were less easily broken. In other words, the addition of rGO increased the structural regularity with more defined pores. During the manufacture of the PCL scaffolds using the TIPS technique (thermally induced phase separation), the introduction of rGO disturbed and modified the growth of the solvent crystals that eventually determine the scaffold structure when the rGO has been sublimated [6].

### 3.2. Crystallization Behavior

Two scans were performed for the thermal characterization of the samples: one from 0 to 100 °C with a heating rate of 10 °C/min, and a second from 100 °C to 0 °C with a cooling rate of 10 °C/min. The experimental results are presented in Table 1. The melting temperature (Tm), melting enthalpy (ΔHm), crystallization enthalpy (ΔHc) and degree of crystallinity (Xc) increased with the addition of rGO. The incorporation of rGO increased the melting temperature from 32 °C for neat PCL to 37.3 °C when incorporating 0.6 wt%, pointing to larger and more perfect crystalline structures [20]. The degree of crystallinity of PCL and its composites was calculated according to Equation (1), where ΔH_m_ is the melting enthalpy, Δ$H_{m}^{0}$ is the melting enthalpy of 100% crystalline PCL taken as 139 Jg^−1^ [21] and X_rGO_ is the fraction of rGO:

(1) $$X_{c}=\frac{{\Delta H}_{m}}{{\Delta H}_{m}^{0}}\;\times \;\left(\frac{1}{1-{\;X}_{\mathrm{rGO}}}\right)\times \;100\;$$

The degree of crystallinity that increased from 35.6% for PCL to 55.9% for PCL/0.6% rGO had a significant influence on the hardness, density, transparency and diffusion [20]. However, the degree of crystallinity is not the only factor in the determination of those properties, as structural unit sizes and molecular orientation must also be considered [20]. The addition of small amounts of rGO changes their thermal properties with which they can be modulated for application in the field of tissue engineering.

### 3.3. Non-Isothermal Melt Crystallization and Kinetics Analysis

Semi-crystalline polymers can crystallize between glass transition temperature (Tg) and the melting temperature (Tm). Depending on the initial state, the crystallization process can be performed in two ways. In the melt crystallization process, the initial state is the molten state and the temperature of the samples are first of all higher than their Tm. In the cold crystallization process, the initial state is the amorphous state and the temperatures of the samples are first of all lower than their Tg [15].

In this section, we studied the crystallization of PCL and its compounds with rGO from the molten state. Specifically, the non-isothermal crystallization processes of PCL, PCL/0.3% rGO, PCL/0.6% rGO and PCL/1% rGO scaffolds were studied at different cooling rates: 5, 10, 15 and 20 °C/min. The investigation was performed on the molten polymer, i.e., the polymer samples were kept at a temperature above their Tm [14]. In all analyses, the porous supports were first cooled to 0 °C, then heated to 100 °C and cooled again to 0 °C at the different cooling rates under study.

**Figure 3:**
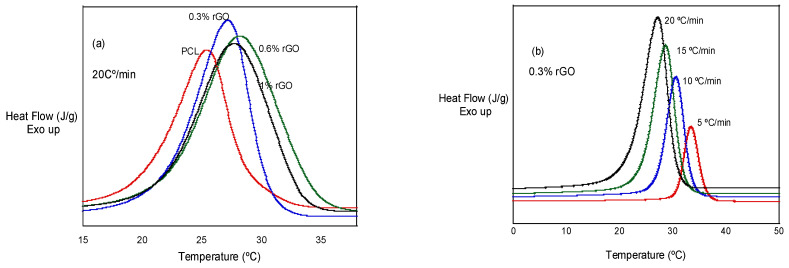
(**a**) Non-isothermal crystallization behavior of PCL and its composites with rGO at 20 °C/min. (**b**) Non-isothermal crystallization behavior of PCL/0.3% rGO at different heating rates.

In Figure 3a, we can observe the effect of rGO additions at a given cooling rate: 20 °C/min. It can be observed that the crystallization peaks are higher and wider with the addition of rGO, with PCL, PCL/0.3% rGO, PCL/0.6% rGO and PCL/1% rGO having temperature ranges of 4.5, 5.0, 6.6 and 6.1 °C, respectively. In Figure 3b, we can observe that the crystallization peak becomes wider and shifts to lower temperatures as the cooling rate increases.

**Figure 4:**
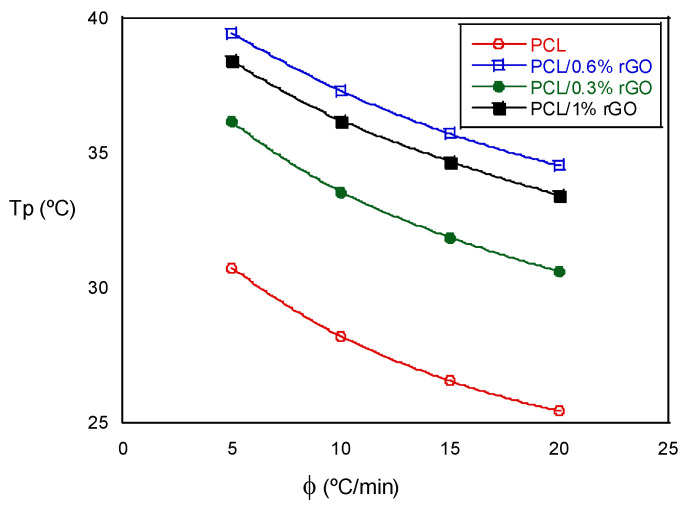
Crystallization peak temperature as a function of the cooling rate for PCL and PCL/rGO scaffolds.

The non-isothermal crystallization temperature, Tp, decreased as the cooling rate increased in the tests with the same composition. When we analyzed the influence of the scaffold composition, we observed that the Tp increased as the amount of rGO increased, an exception being the scaffolds with 1% rGO, the values of which were similar to those with 0.6% rGO. The experimental results indicate that the rGO concentration and cooling rate strongly affect the crystallization process. The non-isothermal crystallization process improved with the cooling rate, but the increase in the rGO slowed down the crystallization process at higher temperatures. These events are best visualized in Figure 4, where the crystallization peak temperature, Tp, is plotted as a function of the cooling rate for all the scaffolds that were investigated. Moreover, the degree of crystallization during cooling increased with rGO content, as deduced from the increase in the crystallization enthalpy (see Table 2). The addition of rGO resulted in nucleation sites, accelerated crystallization kinetics and provided a higher degree of crystallinity. Low cooling rates promoted the nucleation and degree of crystallinity in both the PCL and the PCL/rGO composite scaffolds. Wang et al. [19,22] observed similar behaviors for other polyester poly(l-lactic acid)/graphene oxide nanocomposites, and Qiu et al. [14] for poly(ethylene succinate), although from the amorphous state.

**Figure 5:**
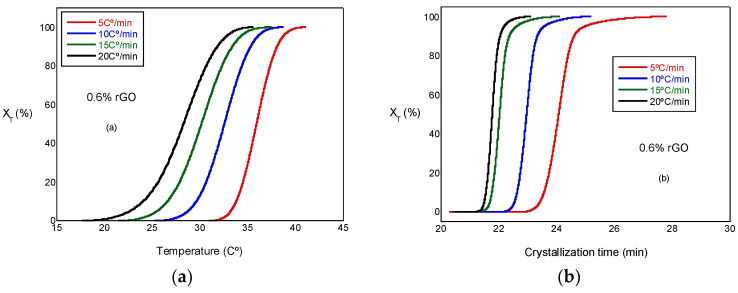
(**a**) Plots of relative crystallinity versus temperature for PCL/0.6% rGO. (**b**) Plot of relative crystallinity versus crystallization time for PCL/0.6% rGO.

The relative crystallinity can be calculated from the integration of data on the crystallization exotherm peaks during non-isothermal melt crystallization as a function of temperature and at a cooling rate of 5–20 °C/min:

(2) $$X_{T}=\int_{\mathrm{To}}^{T\infty }\frac{d\mathrm{Ho}}{dT}\;dT\;\;$$

where T_0_ and T^∞^ are the onset and end of the crystallization temperatures, respectively. Figure 5a presents the relative crystallinity, X_T_, versus the temperature at several cooling rates. These curves have similar sigmoidal shapes. Figure 5b presents the relative crystallinity, X_T_, versus the crystallization time for the PCL and the PCL/rGO scaffolds at several cooling rates. In Figure 5b, the plots can be observed to shift to higher temperature ranges at increasing heating rates. We can observe that the crystallization time becomes shorter at higher cooling rates. Similar behavior was observed in all the test samples, although not all of them are presented here.

The relation between the crystallization time (t) and the corresponding temperature, T, during the non-isothermal melt crystallization can be calculated with the following expression:

(3) $$t=\frac{T-T_{0}}{\Phi }$$

T is the temperature crystallization time t, T_0_ is the temperature at onset of crystallization and Φ is the heating rate.

The crystallization half-time (t_1/2_) is the time needed to reach 50% of the final crystallization of the scaffolds, which can be determined from the graphical representation of relative crystallinity ($X_{T}$) as a function of crystallization time (t) (Figure 5b). The values obtained can be observed in Table 2, where t_1/2_ decreases with the increasing cooling rate for all scaffolds under study and is a little less in the scaffolds with rGO, a result that indicates a minor effect of rGO on nucleation; therefore, the behavior of the PCL in the non-isothermal melt crystallization was increased by the presence of the reinforcement. In other words, rGO acted as a nucleating agent, though with a moderate character. Other authors, such as Wang et al. [19], observed similar behavior. In a previous study conducted with a PLCL/rGO scaffold [22], t_1/2_ increased with the addition of rGO from 10.15 for the PLCL to 13.5 for the compounds with rGO; in this case, the behavior was quite the opposite and the rGO had little or no nucleating effects.

We used the expression of Kanna et al. [23] to calculate the0 crystallization rate coefficient (CRC) with which a direct comparison may be drawn with the crystallization of polymeric systems. CRC represents a change in the cooling rate required to achieve a 1 °C change in the supercooling of the polymer melt. CRC values should be higher for more rapid crystallization systems.

The CRC values (see Table 3) were calculated from the slope of the lines obtained from plotting the cooling rate as a function of the difference between the maximum non-isothermal crystallization temperature (T_p_) and the melting temperature (T_m_). The CRC coefficient increased from 0.89 to 0.99 for samples with rGo. The presence of rGO increased the crystallization rate independently of the rGO concentration that had been added.

**Figure 6:**
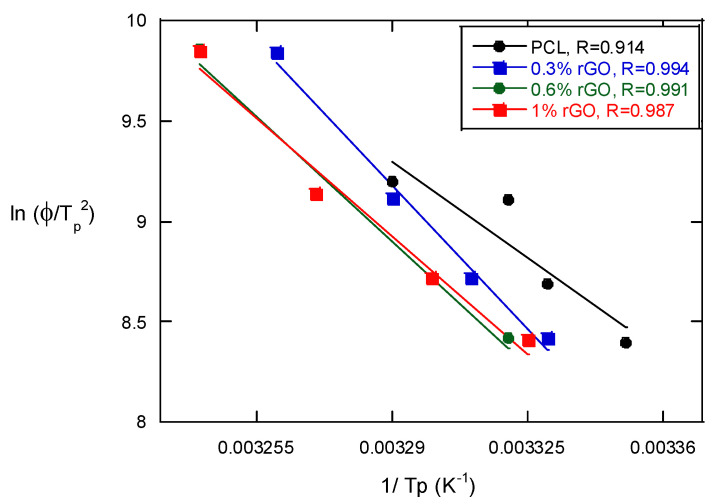
The Kissinger plots of PCL and its composite scaffolds with rGO for the estimation of crystallization activation energy in non-isothermal melt crystallization.

The Kissinger method was used to calculate the activation energy of the non-isothermal melt crystallization process of the PCL scaffolds and their composites with rGO [24]:

(4) $$\frac{d(\ln\left(\frac{\Phi }{T_{p}^{2}}\;\right))}{d(\frac{1}{T_{p}})}=-\frac{\Delta E}{R}\;$$

where Φ is the cooling rate, T_p_ is the crystallization peak temperature, ΔE is the activation energy and R is the universal gas constant. Figure 6 presents the Kissinger equation plots of the PCL scaffolds and the PCL/rGO composite scaffolds. The activation energy can be determined from the slope of the plots and is strongly dependent on the content of rGO. It decreased with the presence of rGO to 333.5 kJ/mol for neat PCL from 220, 200.5 and 198.8 for composites with 0.3, 0.6 and 1% rGO, respectively. These results indicate that the addition of rGO to PCL scaffolds causes heterogeneous nucleation (a lower ΔE) and the increased rGO content causes further heterogeneous nucleation, which varies the transportation capability of the polymer chains during the crystallization process. Wu et al. [25], in their studies of the crystallization of PCL/multiwalled carbon nanotube composites (MWCNTs), observed that the activation energy decreased from 309.8 for pure PCL to 252.3 kJ/mol when the MWCNT concentration was increased with the Avrami method. Although it is another method used to calculate activation energy, we may say that very similar results have been obtained with both the Kissinger and Avrami methods.

## 4. Conclusions

In this study, the morphology, crystallization behavior and non-isothermal crystallinity from the melt state of neat PCL and nanocomposite scaffolds at different rGO contents were investigated. The introduction of rGO in the polymeric solutions disturbed the crystallization of the dissolvent, which resulted in the formation of a more ordered structure with larger and homogeneous pores and thicker walls, i.e., a more isotropic structure. The thermal properties of the porous substrates, such as the degree of crystallinity, increased with the addition of rGO. The crystallization rate coefficient (CRC) increased with the presence of rGO, indicating that the crystallization rate increased. The activation energy calculated with the Kissinger equation decreased with the presence of rGO. The experimental results show that the addition of rGO results in nucleation sites, accelerated crystallization kinetics and provides a higher degree of crystallinity. We can therefore conclude that the rGO acted as a nucleating agent that enhanced the non-isothermal melt crystallization process.

## Figures and Tables

**Table 1:** Thermal properties of PCL and PCL/rGO composite scaffolds.

Samples	T_m_ (°C)	ΔH_m_ (J/g)	ΔH_c_ (J/g)	X_c_ (%)
**PCL**	32.0	49.5	50.9	35.6
**PCL/0.3% rGO**	33.5	63.7	61.2	45.9
**PCL/0.6% rGO**	37.3	77.3	61.0	55.9
**PCL/1% rGO**	36.2	55.7	52.8	40.5

**Table 2:** Summary of relevant thermal parameters for neat PCL and its nanocomposites: heating rate (Φ), non-isothermal peak temperature (Tp), melting temperature (T_m_), crystallization enthalpy (ΔHc) and non-isothermal crystallization half-time (t_1/2_).

Samples	Φ (°C/min)	Tp (°C)	Tm (°C)	ΔHc (J/g)	t_1/2_ (min)
PCL	5	30.76	35.60	51.20	45.92
	10	28.20	32.04	50.89	23.36
	15	26.55	30.38	51.03	15.74
	20	25.43	28.95	50.58	11.92
PLC/0.3% rGO	5	33.45	36.17	61.61	45.54
	10	30.57	33.55	61.16	23.15
	15	28.63	31.87	59.38	15.63
	20	27.18	30.62	59.46	11.84
PLC/0.6% rGO	5	35.71	39.46	63.52	45.07
	10	32.30	37.31	61.06	22.96
	15	29.86	35.71	58.21	15.52
	20	28.14	34.54	56.92	11.77
PLCL/1% rGO	5	35.31	38.42	55.40	45.19
	10	32.17	36.20	52.78	22.99
	15	29.68	34.68	52.26	15.51
	20	27.70	33.44	51.83	11.81

**Table 3:** Crystallization rate coefficient (CRC) parameter for the scaffolds under study.

Sample	CRC
PCL	0.89
PCL/0.3% rGO	0.99
PCL/0.6% rGO	0.98
PCL/1% rGO	0.99

## Data Availability

Not applicable.
